# Hepatitis E Infection in Patients With Inflammatory Bowel Diseases: A Systematic Review and Meta‐Analysis

**DOI:** 10.1111/jvh.70152

**Published:** 2026-02-20

**Authors:** Dionysios Kogias, Georgios Kouklakis, Vasileios Papadopoulos

**Affiliations:** ^1^ First Department of Internal Medicine, Department of Medicine Democritus University of Thrace Alexandroupolis Greece; ^2^ Laboratory of Anatomy, Department of Medicine Democritus University of Thrace Alexandroupolis Greece

**Keywords:** crohn's disease, hepatitis E, immunosuppression, inflammatory bowel disease, ulcerative colitis

## Abstract

Opportunistic infections are increasingly recognised in patients with inflammatory bowel disease (IBD), particularly among those receiving immunosuppressive therapy. Hepatitis E virus (HEV) is a leading cause of acute viral hepatitis worldwide, yet its relevance in IBD remains insufficiently clarified. This systematic review and meta‐analysis aimed to explore the association between HEV infection and IBD. A comprehensive literature search was performed in PubMed/MEDLINE and Google Scholar up to May 2025, identifying all studies reporting HEV infection in patients with ulcerative colitis (UC) or Crohn's disease (CD), with or without a control group. Study quality was assessed using the Joanna Briggs Institute Checklist for Analytical Cross‐Sectional Studies. Six eligible studies encompassing 1316 IBD patients were included in the qualitative and quantitative synthesis. The pooled prevalence of anti‐HEV IgG (HEV‐G) antibodies among IBD patients was 13.5%, though with notable heterogeneity. Anti‐HEV IgM (HEV‐M) and HEV‐RNA positivity rates were significantly lower, at 1.9% and 0.03%, respectively. When compared with the general population, IBD patients exhibited similar HEV‐G and HEV‐M prevalence, and comparable rates were observed between UC and CD subgroups. In contrast, immunocompromised transplant recipients demonstrated markedly higher HEV seropositivity. Sensitivity analyses confined to European cohorts indicated a modest rise in HEV‐G and HEV‐M levels, particularly among patients receiving intensified immunosuppression. Clinically, unexplained elevations of liver enzymes in IBD should prompt consideration of HEV infection. Overall, HEV prevalence in IBD parallels that of the general population; however, severe immunosuppression may predispose to persistent infection or liver‐related complications, warranting routine testing for accurate diagnosis.

**Abbreviations**
ALP: alkaline phosphatase
ALT: alanine transaminase
AST: aspartate transferase
CD: Crohn's disease
CRP: C‐reactive protein
DBIL: direct bilirubin
DILI: drug‐induced liver injury
EASL: European Association for the Study of the Liver
GBD: Global Burden of Disease
GGT: gamma‐glutamyl transferase
HEV: hepatitis E virus
HEV‐G: anti‐HEV IgG antibodies
HEV‐M: anti‐HEV IgM antibodies
HEV RNA: hepatitis E virus ribonucleic acid
IBD: inflammatory bowel disease
NAT: nucleic acid tests
PSC: primary sclerosing cholangitis
RBV: ribavirin
RNA: ribonucleic acid
RT‐PCR: real‐time polymerase chain reaction
SVR: sustained virological response
TBIL: total bilirubin
UC: ulcerative colitis

## 1 Introduction

Hepatitis E virus (HEV) is a single‐stranded RNA virus classified within the Hepeviridae family and is a primary cause of acute hepatitis, primarily transmitted via the faecal‐oral route. While historically recognised as a major cause of epidemic hepatitis in developing countries, HEV has recently emerged as a global public health concern. Phylogenetic analysis categorises HEV into eight genotypes (HEV1–8). HEV1 and HEV2 are exclusive to humans and are commonly linked to epidemics in developing regions due to inadequate hygiene and sanitation. HEV3 and HEV4 are zoonotic, primarily infecting animals such as pigs, wild boars, and deer, with human transmission occurring through the consumption of contaminated meat. These genotypes are prevalent in industrialised nations and are responsible for sporadic and clustered cases of hepatitis E. HEV5 and HEV6 have been identified only in wild boars, while HEV7 and HEV8 have been detected in camels. Notably, HEV7 has been reported in a small number of immunocompromised individuals following the consumption of camel meat and milk [1, 2].

The majority of HEV infections are asymptomatic or present with mild, self‐limiting symptoms, with most patients recovering without medical intervention [3]. However, progression to chronic hepatitis E—defined as the inability to clear the virus within three months of initial infection—is uncommon and occurs primarily in immunocompromised individuals. Chronic HEV infection is most frequently associated with HEV3 and HEV4, with sporadic cases linked to HEV7 [4, 5, 6]. Common risk factors for chronicity include immunosuppressive conditions, such as those affecting patients undergoing haemodialysis [7], recipients of solid organ transplants, and individuals with haematological malignancies or autoimmune disorders receiving immunosuppressive therapy [8, 9].

Patients with inflammatory bowel disease (IBD) have an increased risk of infections due to immune system alterations resulting from the disease's pathophysiology and the use of immunomodulatory medications. Elevated liver enzyme levels are commonly observed in individuals with IBD and are primarily associated with nonalcoholic fatty liver disease, primary sclerosing cholangitis (PSC), or drug‐induced liver injury (DILI) [10, 11, 12]. Several studies have investigated the prevalence of HEV infection among IBD patients, some with contradictory results [13, 14, 15, 16, 17, 18, 19, 20, 21, 22, 23, 24, 25, 26].

The present systematic literature review and meta‐analysis was conducted with the aim of providing further evidence about the prevalence of acute and chronic HEV infections in patients with IBD by identifying all relevant studies and summarising their results.

## 2 Materials and Methods

### 2.1 Literature Search

A systematic literature review was conducted using the PubMed/MEDLINE database until May 7, 2025 to identify all studies that reported HEV infection in patients with UC and CD. The SciELO and Google Scholar databases were used as an additional pool of published data. The search was performed independently by two authors (DK and VP). No automation tool was used. The PRSIMA guidelines were considered for reporting [27].

### 2.2 Study Selection

The review was conducted using a search strategy that included the terms [inflammatory bowel disease] AND [hepatitis E virus]; [inflammatory bowel disease] AND [HEV]; [ulcerative colitis] AND [hepatitis E virus]; [ulcerative colitis] AND [HEV]; [crohn] AND [hepatitis E virus]; [crohn] AND [HEV]. No software was used for study retrieval.

A study was considered eligible if: (a) referred to adult patients; (b) reported at least one outcome measure; and (c) contained at least an English abstract. Exclusion criteria included: (a) publications in forms of case reports, narrative reviews, and editorials. The selection was performed independently by two authors (DK and VP). Any dispute was resolved by a third author (GK).

### 2.3 Outcome Measures

PICO (P—Populations/People/Patient/Problem: Patients suffering from any type of IBD (UC and CD), sex‐ and age‐adjusted healthy individuals (healthy controls), and immunocompromised patients (immunocompromised controls); I—Intervention(s): HEV infection; C—Comparison: (i) prevalence of HEV IgG and IgM antibodies and HEV RNA positive value among patients suffering from CD compared with UC; (ii) prevalence of HEV‐G, HEV‐M, and HEV RNA positive value among IBD patients and healthy controls; (iii) prevalence of HEV IgG and IgM antibodies and HEV RNA positive value among IBD patients and immunocompromised controls; O—Outcome: OR; percentage) worksheet and search strategy.

HEV‐related outcome in IBD patients measures data were compared with the meta‐analysis of Li et al. [28] for general population, while data from the meta‐analysis of Buescher et al. were used to compare outcome measures between IBD patients and immunocompromised patients [29].

### 2.4 Data Acquisition

For every included study, all relevant data concerning the name of the first author, the year that the study was conducted, the country that the publication derived from, the number of patients included, the male ratio of the patients, the mean age and the standard deviation of the patients, and the HEV‐G, HEV‐M, and HEV‐RNA positives among analysed samples were collected. In any case that IBD patients were reported, discrimination between CD and UC was recorded.

### 2.5 Quality Assessment

The Johanna Briggs Institute (JBI) Checklist for Analytical Cross‐Sectional Studies was used [30]. In detail, eight elements (domains) were assessed by the respective questions, namely, Q1: “Were the criteria for inclusion in the sample clearly defined?”; Q2: “Were the study subjects and the setting described in detail?”; Q3: “Was the exposure measured in a valid and reliable way?”; Q4:“Were objective, standard criteria used for measurement of the condition?”; Q5: “Were confounding factors identified?”; Q6: “Were strategies to deal with confounding factors stated?”; Q7: “Were the outcomes measured in a valid and reliable way?”; Q8: “Was appropriate statistical analysis used?” For each element, a response was recorded as “Yes” (Y), “No” (N), “Unclear (U),” and “Not Applicable” (NA) accordingly. A study was considered of acceptable quality if negative responses were ≤ 25% of the total elements assessed.

### 2.6 Evidence Synthesis

Data were analysed using STATA 19 and RevMan 5.3. Means and standard deviations (SDs) were combined using an online tool (https://www.statstodo.com/CombineMeansSDs.php, accessed 7 May 2025), while estimates based on sample size, median, range, and interquartile range were derived using another online tool (https://www.math.hkbu.edu.hk/~tongt/papers/median2mean.html, accessed 7 May 2025). Heterogeneity was assessed using the Q test (*p* < 0.10 indicating significance) and the I^2^ statistic (≤ 25%: insignificant; 26%–50%: low; 51%–75%: moderate; > 75%: high). Prevalence and proportion data were pooled implementing Freeman–Tukey double arcsine transformation. A random‐effects model was used when I^2^ > 50, else a fixed‐effects model was preferred. A Galbraith plot identified potential outliers. Publication bias was evaluated via funnel plot symmetry, supported by Egger's and Begg's tests, with the trim‐and‐fill method used to adjust for missing studies. Sensitivity analysis was conducted using a leave‐one‐out approach to examine the impact of individual studies on heterogeneity. Subgroup analysis was further used to investigate potential sources of heterogeneity.

## 3 Results

### 3.1 Literature Search / Study Selection / Quality Assessment

In total, 77 studies were identified. After removal of the 20 duplicates, the remaining 57 studies were considered for eligibility by title/abstract, and 13 were further judged based on full text. Six studies fulfilled the eligibility criteria and were qualitatively and quantitatively analysed (Table 1, Figure SS1). The quality assessment is analytically presented in Supplementary Table S1.

**TABLE 1:** Included studies; HL‐IT: High‐level immunosuppression therapy rate.

Study^†^	Region	HL‐IT	Patients (CD/UC)	Male ratio	Mean Age (SD)	Anti‐HEV IgG (CD/UC)	Anti‐HEV IgM (CD/UC)	HEV‐RNA (CD/UC)
Senosiain, 2015, [13]	Spain	0.517	87 (50/36)^‡^	0.552	44.7 (16.0)	1/80 (1/0)	2/75 (2/0)	0/46
Garrido, 2019, [14]	Portugal	0.823	62 (57/5)	0.516	48.8 (14.6)	22/62	—	—
Hoffmann, 2020, [15]	Germany	0.841	478 (328/150)	0.477	44.3 (10.4)	94/478 (57/37)	—	0/478
Grigas, 2021, [16]	Lithuania	0.207	203 (47/156)	0.581	40.0 (15.0)	25/203 (6/19)	13/203 (4/9)	1/203 (0/1)
Kounis, 2023, [17]	France	1.000	488 (327/161)	0.508	41.7 (14.7)	69/488 (46/21)	3/488	0/488
Santos, 2023, [18]	Brazil		38			3/38	0/38 (0/0)	

^†^ All studies were cross‐sectional.

^‡^ One patient had undetermined colitis.

### 3.2 Synthesis

**FIGURE 1:**
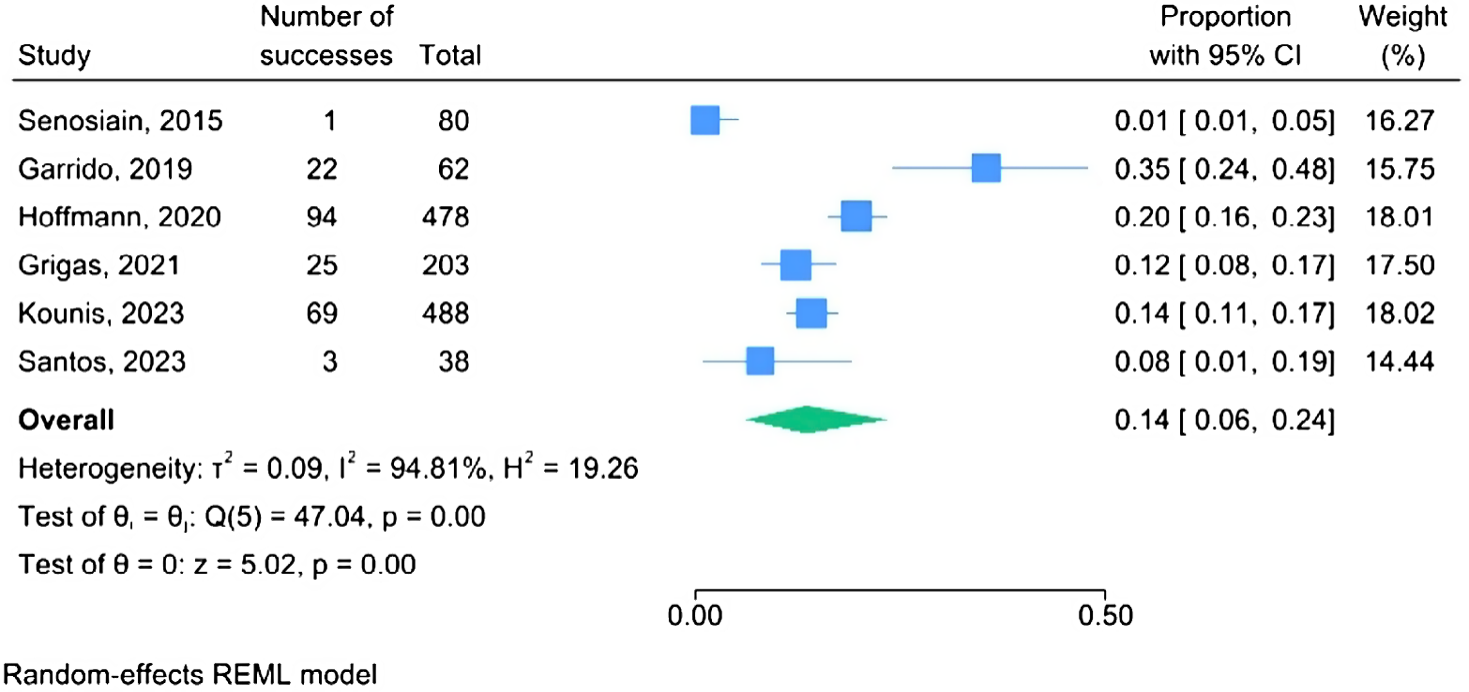
HEV‐G prevalence among IBD patients; forest plot.

Among IBD patients, the pooled prevalence of HEV‐G antibodies was 13.5% (95% CI: 5.8–23.7) with considerable heterogeneity (I^2^ = 94.8%) (Figure 1). Moreover, the pooled prevalence of HEV‐M and HEV‐RNA was 1.9% (95% CI: 0.0–5.5; I^2^ = 79.2%) (Figure SS2) and 0.03% (95% CI: 0.00–0.20; I^2^ = 0.0%), respectively (Figure SS3).

When IBD patients are compared with the general population, using a pooled estimate from 287 studies [28], they present comparable HEV‐G prevalence (13.5% (95% CI: 5.8–23.7) vs. 12.47% with 95% CI: 10.42–14.67; RR 1.08 with 95% CI: 0.55–2.14; *p* = 0.83). Similarly, the prevalence of HEV‐M in IBD patients is 1.9% (95% CI: 0.0–5.5), when compared to the general population (1.47%; 95% CI: 1.14–1.85; pooled data from 98 studies), yields an RR of 1.29 with 95% CI: 0.30–5.58 (*p* = 0.76) for acute HEV infection. Lastly, HEV‐RNA is comparable (*p* = 0.06) between IBD patients (0.03%; 95% CI: 0.00–0.20) and healthy individuals (0.20%; 95% CI: 0.15–0.25).

**FIGURE 2:**
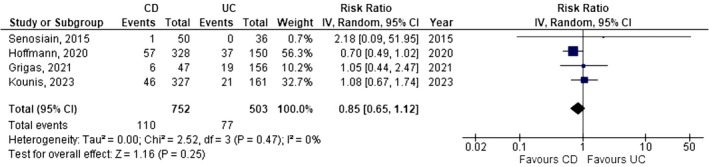
Comparison between HEV‐G prevalence among CD and UC patients; forest plot.

Of note, the prevalence of HEV‐G among CD (11.5%; 95% CI: 5.6–19.1; Figure SS4) and UC (11.0%; 95% CI: 2.6–23.7; Figure SS5) patients is comparable (RR 0.85; 95% CI: 0.65–1.12; I^2^ 0%; *p* = 0.25; Figure 2). However, concerning HEV‐M and HEV‐RNA, data are insufficient for direct comparison.

Among transplanted immunocompromised patients, HEV‐G are more prevalent (23.4%–36.5%) than IBD patients (16.8; *p* < 0.05). Similarly, HEV‐RNA is strikingly more prevalent among transplanted patients (0.95%–2.08%) than among IBD patients (0.08; *p* < 0.001). In detail, HEV‐G prevalence comparison between IBD patients and immunocompromised patients and relevant data regarding HEV‐RNA are provided in Table 2.

**TABLE 2:** A, RR of anti‐HEV IgG prevalence in immunocompromised vs. IBD patients; B, RR of active viremia (HEV‐RNA positive) in immunocompromised vs. IBD patients.

Group	Prevalence	RR; 95% CI (vs. IBD)	*p*
A			
IBD patients (pooled)	13.5 (5.8–23.7)	1.00 (reference)	
Kidney‐transplanted patients	33.9 (24.0–45.6)	2.51 (1.48–3.78)	< 0.001
Liver‐transplanted patients	27.4 (18.3–38.9)	2.03 (1.33–3.10)	0.001
Heart‐transplanted patients	36.5 (20.2–56.6)	2.70 (1.48–4.94)	0.001
Lung‐transplanted patients	32.5 (11.8–63.3)	2.41 (0.98–5.91)	0.055
Stem Cell‐transplanted patients	25.6 (13.2–43.9)	1.90 (0.97–3.70)	0.061
B			
IBD patients (pooled)	0.03 (0.00–0.20)	1.00 (reference)	
Kidney‐transplanted patients	1.17 (0.68–2.00)	39.0 (5.2–293)	< 0.001
Liver‐transplanted patients	0.95 (0.56–1.63)	31.7 (4.2–239)	0.001
Lung‐transplanted patients	2.08 (0.69–6.08)	69.3 (8.6–558)	< 0.001
Stem Cell‐transplanted patients	1.28 (0.44–3.62)	42.7 (5.3–342)	< 0.001

### 3.3 Heterogeneity Analysis

Applying a leave‐one‐out influence analysis suggests that none of the individual studies significantly influenced the pooled proportion when excluded (Figure SS6).

Performing a sensitivity analysis by restricting results to European countries, the pooled prevalence of HEV‐G was 14.6% (95% CI: 5.5–27.0), while that of HEV‐M was 2.6% (95% CI: 0.0–7.9) (Figure SS7 and Figure SS8). Based on Europe‐specific prevalence of HEV‐G (9.31%; 95% CI: 7.35–11.48; 97 studies), and HEV‐M (0.79%; 95% CI: 0.30–1.51; 26 studies), as assessed by Li et al., IBD patients presented an increased, though nonsignificant, RR to be positive for HEV‐G (RR 1.57; 95% CI: 0.48–3.67; *p* = 0.34) and HEV‐M (RR 3.29; 95% CI: 0.00–26.33; *p* = 0.37).

**FIGURE 3:**
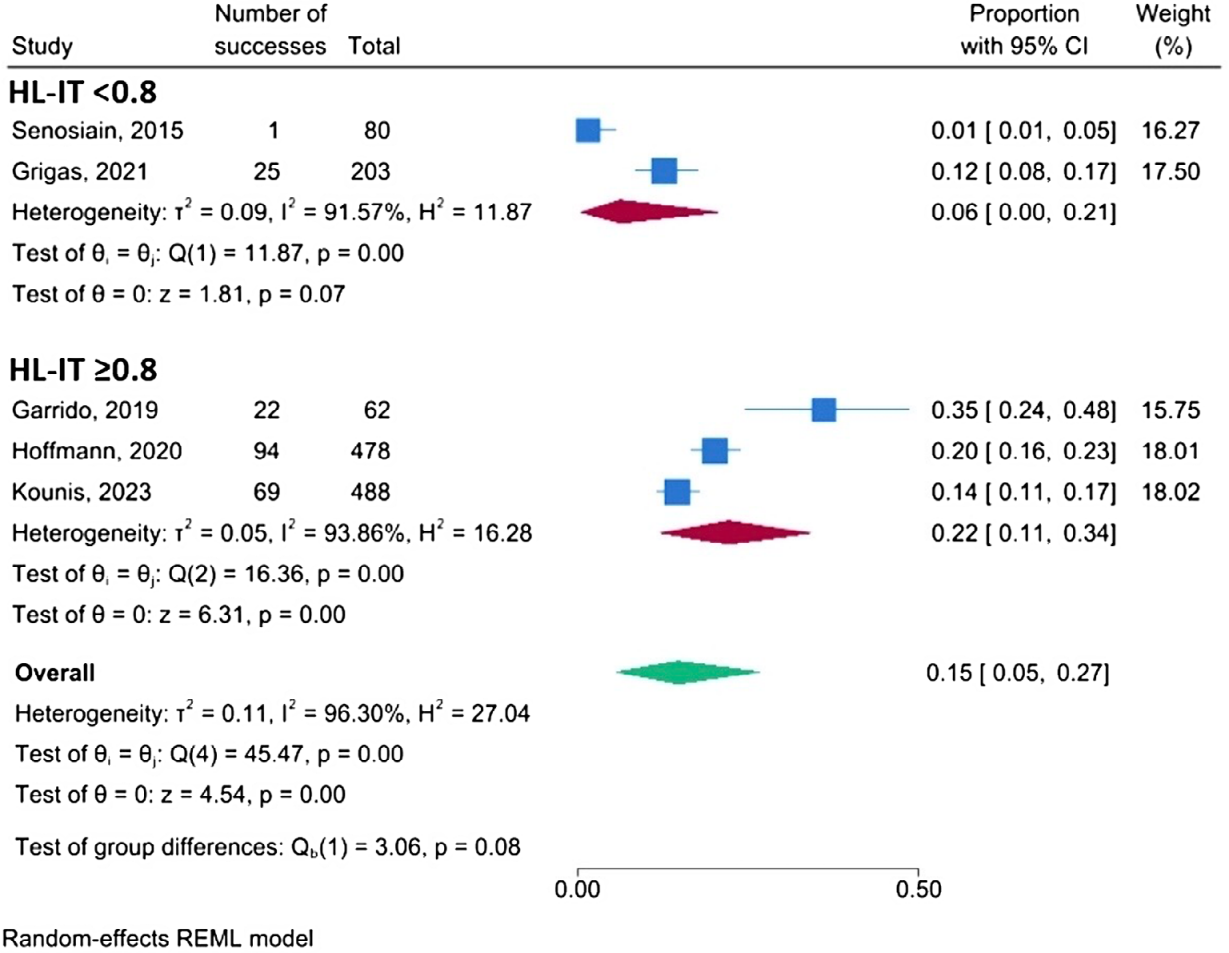
Subgroup analysis according to high‐level immunosuppression therapy rate (HL‐IT); forest plot.

Furthermore, when a subgroup analysis according to high‐level immunosuppression therapy rate (HL‐IT) was performed, IBD patients who administered HL‐IT ≥ 80% had increased HEV‐G when compared to those with < 80% (21.6%; 95% CI: 11.3–34.0 vs. 5.8%; 95% CI: 0.0–20.6, Q‐test *p* = 0.08) (Figure 3).

### 3.4 Bias Assessment

The Galbraith plot does not suggest strong outliers (Figure SS9). No publication bias was detected; there was no apparent funnel plot asymmetry. Moreover, applying trim‐and‐fill analysis, no imputed studies were observed (Figure SS10). Additionally, both the Egger's and the Begg's test yielded non‐significant results (*p* = 0.805 and 1.000, respectively).

## 4 Discussion

It has been estimated that one‐third of the global population, representing over two billion people, live in HEV endemic areas at risk of infection [28]. In fact, the true burden of hepatitis E remains largely unknown. To the best of our knowledge, this is the first systematic review and meta‐analysis concerning the relation between HEV infection and IBD and provides a comprehensive synthesis of the available evidence regarding the prevalence of HEV among patients with IBD. Our results suggest that the pooled seroprevalence of anti‐HEV IgG antibodies in IBD patients is 13.5%, which is comparable to that observed in the general population, with no statistically significant difference (RR 1.08; *p* = 0.83). This finding suggests that IBD itself may not confer an increased risk of HEV exposure or infection.

Similarly, the prevalence of markers of acute infection, including anti‐HEV IgM antibodies and HEV‐RNA, did not significantly differ between IBD patients and healthy controls. HEV‐M was detected in 1.9% of IBD patients, compared to 1.47% in the general population, with a non‐significant RR of 1.29 (*p* = 0.76). HEV‐RNA, indicative of active infection, was exceptionally rare among IBD patients (0.03%) and not significantly different from the general population (0.20%; *p* = 0.06) [28].

Importantly, subgroup analysis revealed no significant differences in HEV‐G prevalence between CD and UC patients (11.5% vs. 11.0%, respectively; RR 0.85; *p* = 0.25), suggesting that disease subtype does not appear to influence HEV seropositivity. However, data were insufficient to make robust comparisons for HEV‐M and HEV‐RNA across CD and UC subgroups.

Immunosuppressive therapy offers significant therapeutic benefits in the management of IBD. Nevertheless, prolonged use of such treatments may be linked to an elevated risk of infections [31]. Consequently, individuals with IBD receiving corticosteroids, immunosuppressants, or biological agents should be regarded as immunocompromised and susceptible to opportunistic infections [32]. The prevalence and types of infections—including Hepatitis E—among IBD patients vary geographically, largely influenced by local endemic conditions. Analysing all six studies that were included in the present study, the majority of patients who had HEV infection received immunosuppressive therapy either with biologic agents or corticosteroids. However, several patients who had no previous treatment with immunomodulatory regimens were also infected by HEV. That indicated that, although the contribution of immunosuppressive therapy to infection susceptibility in IBD remains a subject of debate, infections represent a substantial burden for affected patients, and immunosuppression appears to play a contributory role [33, 34, 35].

When comparing IBD patients to other immunocompromised populations, particularly transplant recipients, stark differences emerged. HEV‐G seroprevalence and HEV‐RNA positivity were significantly higher in transplant patients (23.4%–36.5% and 0.95%–2.08%, respectively) compared to IBD patients (16.8% and 0.08%, respectively; *p*‐values < 0.05 and < 0.001) (Table 2) [29]. These differences likely reflect the more profound and prolonged immunosuppression among transplant recipients compared to IBD populations. Considering potential risk factors for HEV infection—such as travel history, prior blood transfusions, and the consumption of seafood, wild boar, deer or pork—the available data remain limited. Specifically, Senosiain et al. reported that two of three HEV‐infected IBD patients had a history of consuming seafood and undercooked pork [13]. Among the broader IBD cohort, 10 patients resided in rural areas, 29 had a history of travel, and 15 had received previous transfusions. Of the three patients infected with HEV, one had travelled to Morocco, and another had a history of transfusion. In a separate study, Kounis et al. investigated HEV infection in IBD patients undergoing immunosuppressive therapy and identified raw seafood consumption as the only statistically significant risk factor for HEV exposure [17].

Our heterogeneity analysis highlighted substantial between‐study variability, particularly for HEV‐G, although sensitivity analyses, including a leave‐one‐out approach, confirmed the robustness of our estimates (Figure SS6). Notably, restricting analyses to European cohorts yielded a slightly higher pooled prevalence of HEV‐G (14.6%) and HEV‐M (2.6%) among IBD patients. Yet, even in this region‐specific analysis, risk ratios comparing IBD patients with the general population remained nonsignificant (HEV‐G: RR 1.57, *p* = 0.34; HEV‐M: RR 3.29, *p* = 0.37), likely reflecting overlapping confidence intervals and limited power (Figure SS7 and Figure SS8).

Interestingly, patients undergoing high‐level immunosuppressive therapy (HL‐IT ≥ 80%) exhibited numerically higher HEV‐G prevalence (21.6%) compared to those with less frequent HL‐IT exposure (5.8%), although this difference did not reach conventional statistical significance (*p* = 0.08) (Figure 3). These findings may warrant further investigation as they hint at a possible dose–response relationship between immunosuppression intensity and HEV exposure or reactivation risk.

Furthermore, an analysis of the eight case reports available in the literature revealed that seven patients were infected with genotype 3—commonly found in developed countries—while only one patient was infected with genotype 1, following travel to India [19, 20, 21, 22, 23, 24, 25, 26]. All eight patients were residing in Europe (seven in France and one in the Netherlands). Among them, two were transplant recipients undergoing immunosuppressive therapy, four were being treated with biologic agents, one was receiving azathioprine, and one was on mesalazine monotherapy. Regarding risk factors for HEV infection in these patients, the available data are limited. One patient infected with genotype 1 had a history of travel to India, while another patient infected with genotype 3 had consumed undercooked meat. Additionally, two patients had a history of prior blood transfusions.

Taken together, risk factors for HEV infection appear to play a significant role in determining susceptibility to the disease. Current prophylactic guidelines remain inadequate in preventing all clinically significant infections, particularly opportunistic ones. As a result, careful and continuous monitoring is crucial in the management of IBD patients undergoing immunosuppressive therapy.

Beyond seroprevalence, the clinical relevance of HEV infection in IBD lies primarily in its hepatic manifestations. Several studies have reported that HEV infection in IBD patients may lead to varying degrees of liver injury, ranging from transient elevation of transaminases to clinically significant hepatitis [17, 24, 36]. In most cases, biochemical abnormalities such as raised ALT, AST, and GGT resolve spontaneously within a few weeks, consistent with the self‐limiting nature of HEV infection in immunocompetent individuals [4]. However, in immunosuppressed IBD patients—particularly those treated with azathioprine, methotrexate, corticosteroids, or anti‐TNF agents—HEV may cause prolonged cholestasis, chronic hepatitis, or progressive hepatic fibrosis [17, 37]. Chronic HEV infection, although uncommon, has been increasingly described in this population, emphasising the potential for viral persistence when immune control is impaired. Histopathological findings in such cases include portal inflammation, interface hepatitis, and variable degrees of fibrosis resembling autoimmune or drug‐induced liver injury [36]. Because abnormal liver function tests are frequent in IBD and often attributed to hepatotoxic medication or primary sclerosing cholangitis, HEV infection should be considered in the differential diagnosis of any unexplained hepatic enzyme elevation. Systematic testing for HEV antibodies and HEV‐RNA could prevent diagnostic delays and unnecessary treatment discontinuations. Furthermore, as liver‐related morbidity may complicate the clinical course of IBD, early recognition and supportive management are crucial. The limited number of studies reporting detailed hepatic outcomes highlights an important research gap. Future prospective investigations should assess the natural history of HEV‐associated liver dysfunction in IBD and evaluate whether antiviral therapy or temporary withdrawal of immunosuppressive agents can optimise hepatic recovery and prevent chronic evolution.

Of note, there are some limitations of our study. First, the data analysed were derived from a limited number of cross‐sectional studies. Second, the substantial heterogeneity in HEV‐G estimates limits the precision of our pooled prevalence and suggests variability in study design, population characteristics, or serological assays. Second, while subgroup analyses were informative, some categories—particularly those concerning HEV‐M and HEV‐RNA—were limited by small sample sizes and underpowered comparisons. Third, geographic variation in HEV epidemiology and differential exposure risks may confound comparisons between IBD and general or immunocompromised populations. We were unable to perform a more detailed analysis of regional prevalence, genotype‐specific disease burden, or clinical outcomes as such information is currently available only through individual case reports.

In conclusion, the prevalence of HEV infection among IBD patients is similar to that of the general population, with no significant differences observed for HEV‐G, HEV‐M, or HEV‐RNA. There is no detectable difference in HEV seroprevalence between CD and UC, and any variation in HEV prevalence among IBD patients is most likely attributable to the degree of immunosuppressive therapy. This is further supported by the markedly higher HEV prevalence observed in transplant recipients and IBD patients receiving high‐level immunosuppression. These findings suggest that while IBD itself does not increase HEV risk, intensified immunosuppressive treatment may predispose to higher HEV exposure or reactivation. Clinicians should be aware of this potential risk in managing immunosuppressed IBD patients, particularly in regions with higher HEV prevalence. Routine HEV testing is recommended in unexplained hepatic dysfunction, as timely diagnosis prevents mismanagement and reduces the risk of persistent infection or progressive liver damage.

## Funding

The authors have nothing to report.

## Conflicts of Interest

The authors declare no conflicts of interest.

## Supporting information


**Data S1:** Supplementary Legends.

**Figure S1:** PRISMA 2020 flow diagram.

**Figure S2:** HEV‐M prevalence among IBD patients; forest plot.

**Figure S3:** HEV‐RNA prevalence among IBD patients; forest plot.

**Figure S4:** HEV‐G prevalence among CD patients; forest plot.

**Figure S5:** HEV‐G prevalence among UC patients; forest plot.

**Figure S6:** Leave‐one‐out influence analysis; forest plot.

**Figure S7:** Sensitivity analysis: European‐specific HEV‐G prevalence among IBD patients; forest plot.

**Figure S8:** Sensitivity analysis: European‐specific HEV‐M prevalence among IBD patients; forest plot.

**Figure S9:** Galbraith plot.

**Figure S10:** Funnel plot; no apparent asymmetry is observed, and no imputed studies are detected applying trim‐and‐fill analysis.

**Table S1:** Quality assessment of the included studies.

## Data Availability

The data that support the findings of this study are available from the corresponding author upon reasonable request.
