# Supplementary material for: Hepatitis E Infection in Patients With Inflammatory Bowel Diseases: A Systematic Review and Meta‐Analysis
Source: J Viral Hepat. 2026 Feb 20;33(3):e70152. doi: 10.1111/jvh.70152 (PMC12923650; doi:10.1111/jvh.70152)

**Supplementary Figure 1.** PRISMA 2020 flow diagram.

**
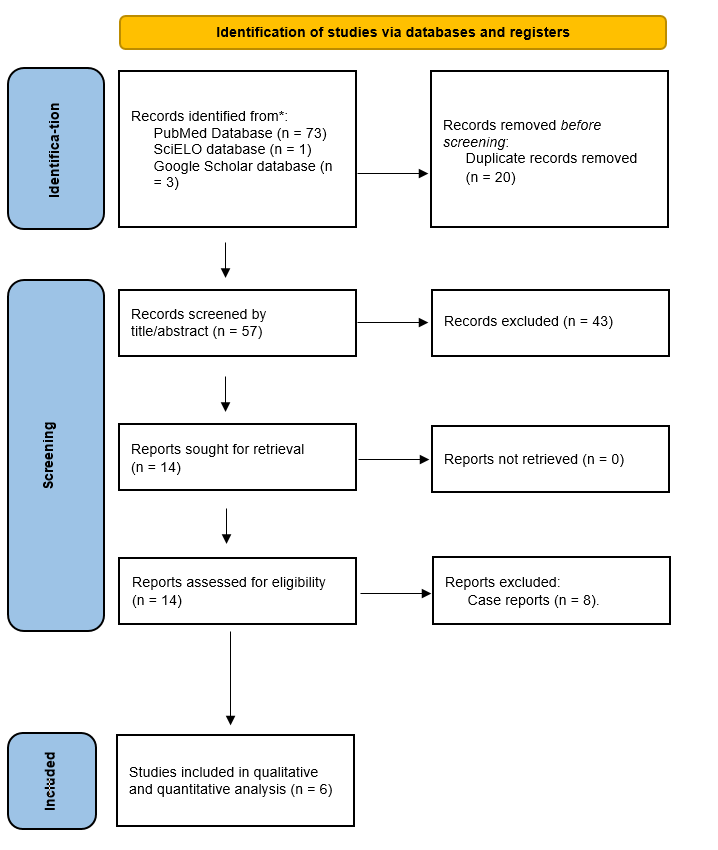
**

**Supplementary Figure 2.** HEV-M prevalence among IBD patients; forest plot.

**
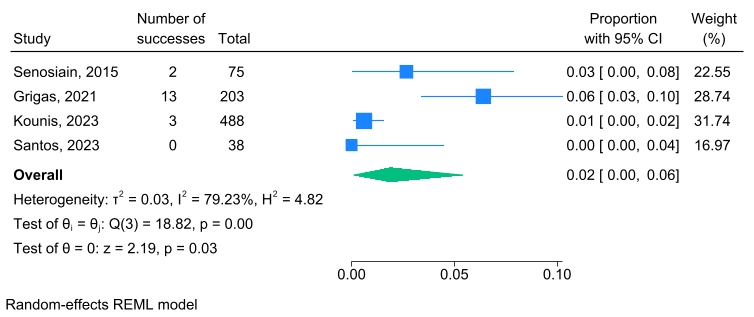
**

**Supplementary Figure 3.** HEV-RNA prevalence among IBD patients; forest plot.

**
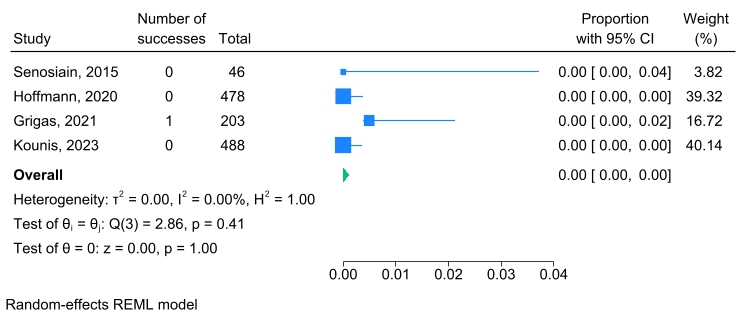
**

**Supplementary Figure 4.** HEV-G prevalence among CD patients; forest plot.

**
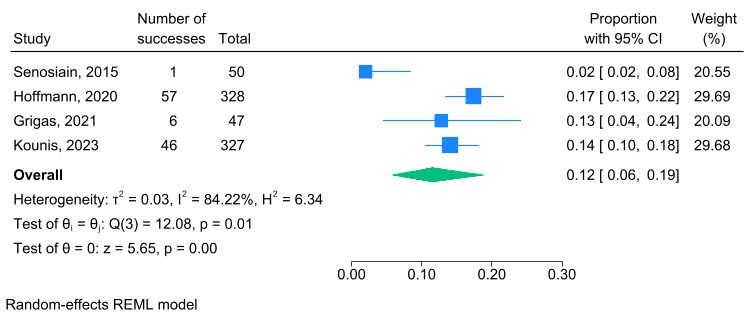
**

**Supplementary Figure 5.** HEV-G prevalence among UC patients; forest plot.

**
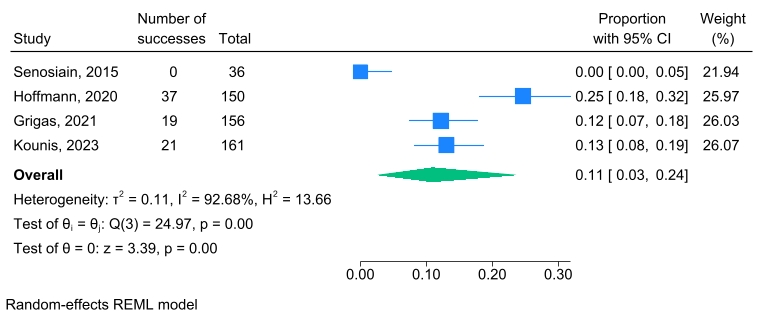
**

**Supplementary Figure 6.** Leave-one-out influence analysis; forest plot.


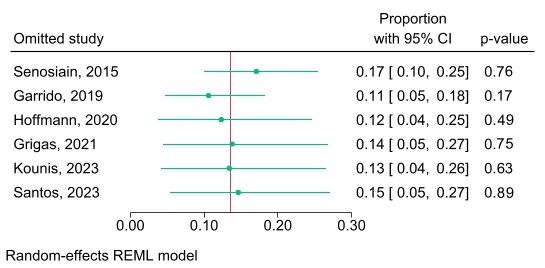


**Supplementary Figure 7.** Sensitivity analysis: European-specific HEV-G prevalence among IBD patients; forest plot.

**
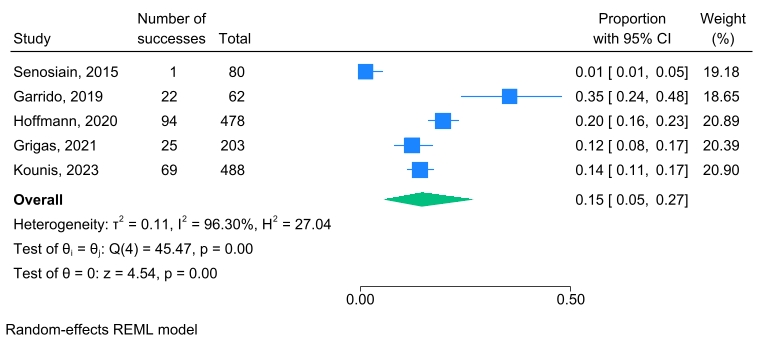
**

**Supplementary Figure 8.** Sensitivity analysis: European-specific HEV-M prevalence among IBD patients; forest plot.

**
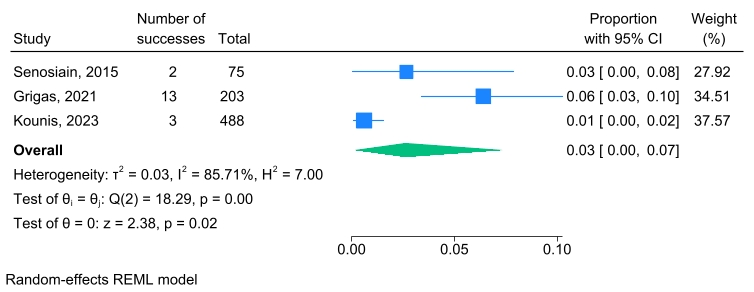
**

**Supplementary Figure 9.** Galbraith plot.


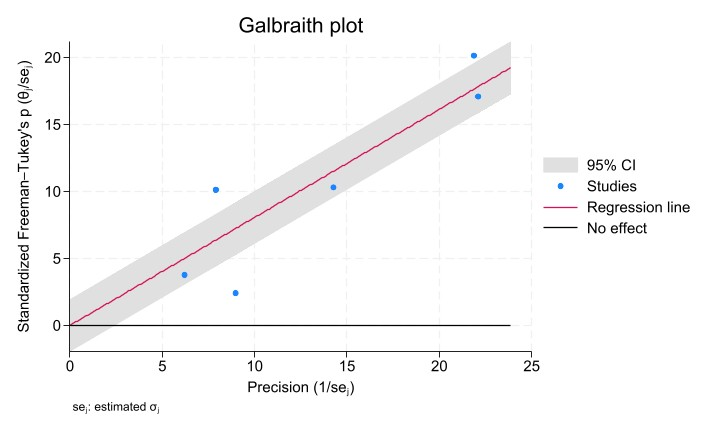


**Supplementary Figure 10.** Funnel plot; no apparent asymmetry is observed, and no imputed studies are detected applying trim-and-fill analysis.


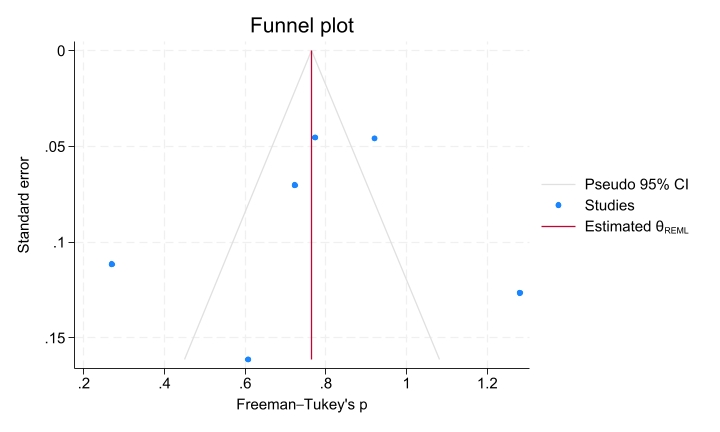

Supplement: Supplementary file 1 — Data S1: Supplementary Legends. [file JVH-33-0-s008.docx]
