# Supplementary figures and images for: Hepatitis E Infection in Patients With Inflammatory Bowel Diseases: A Systematic Review and Meta‐Analysis
Source: J Viral Hepat. 2026 Feb 20;33(3):e70152. doi: 10.1111/jvh.70152 (PMC12923650; doi:10.1111/jvh.70152)

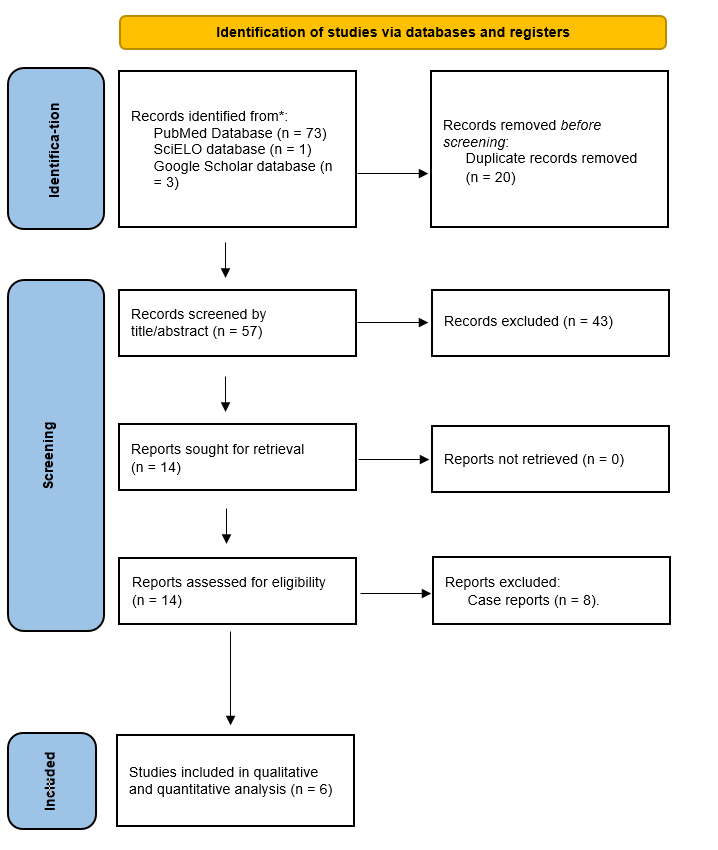

Supplement: Supplementary file 2 — Figure S1: PRISMA 2020 flow diagram. [file JVH-33-0-s006.tiff]

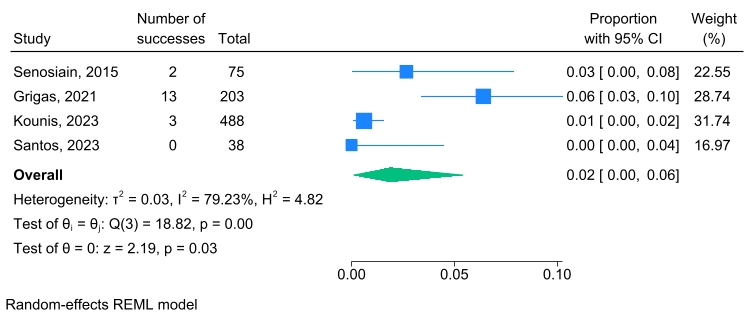

Supplement: Supplementary file 3 — Figure S2: HEV‐M prevalence among IBD patients; forest plot. [file JVH-33-0-s007.tiff]

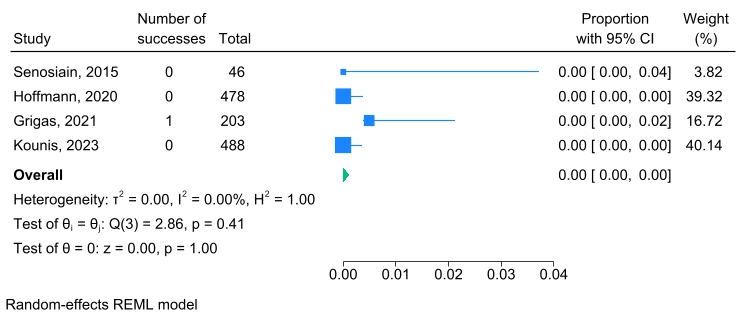

Supplement: Supplementary file 4 — Figure S3: HEV‐RNA prevalence among IBD patients; forest plot. [file JVH-33-0-s010.tiff]

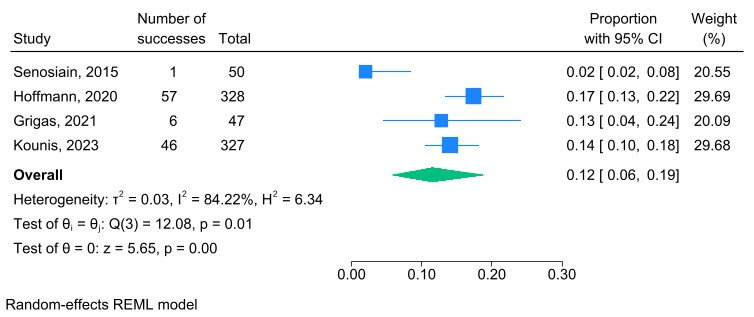

Supplement: Supplementary file 5 — Figure S4: HEV‐G prevalence among CD patients; forest plot. [file JVH-33-0-s001.tiff]

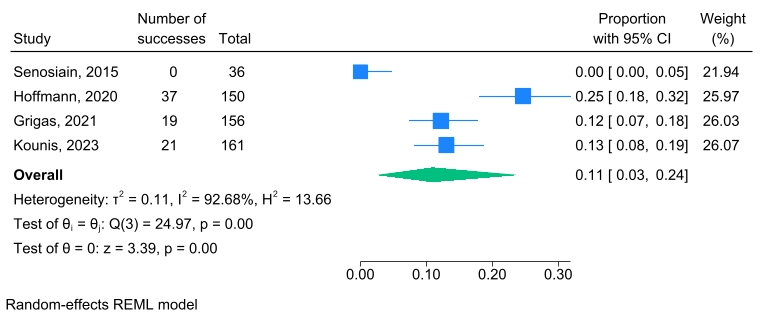

Supplement: Supplementary file 6 — Figure S5: HEV‐G prevalence among UC patients; forest plot. [file JVH-33-0-s012.tiff]

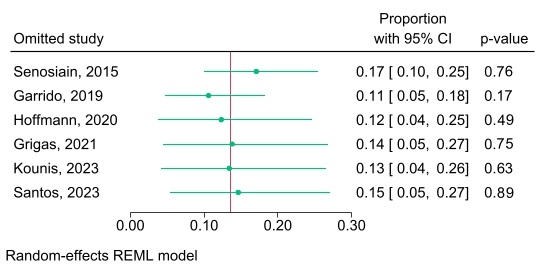

Supplement: Supplementary file 7 — Figure S6: Leave‐one‐out influence analysis; forest plot. [file JVH-33-0-s009.tiff]

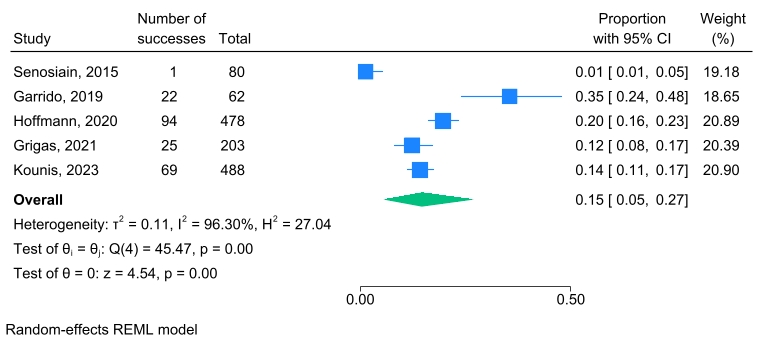

Supplement: Supplementary file 8 — Figure S7: Sensitivity analysis: European‐specific HEV‐G prevalence among IBD patients; forest plot. [file JVH-33-0-s005.tiff]

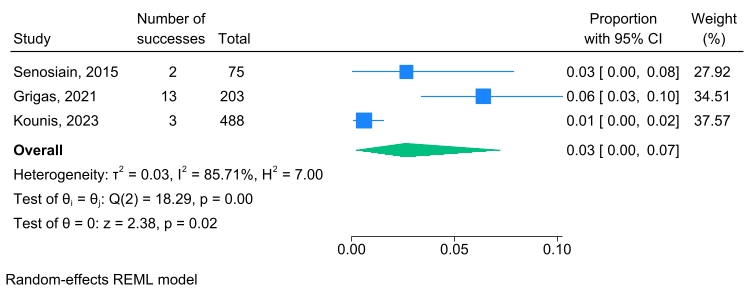

Supplement: Supplementary file 9 — Figure S8: Sensitivity analysis: European‐specific HEV‐M prevalence among IBD patients; forest plot. [file JVH-33-0-s002.tiff]

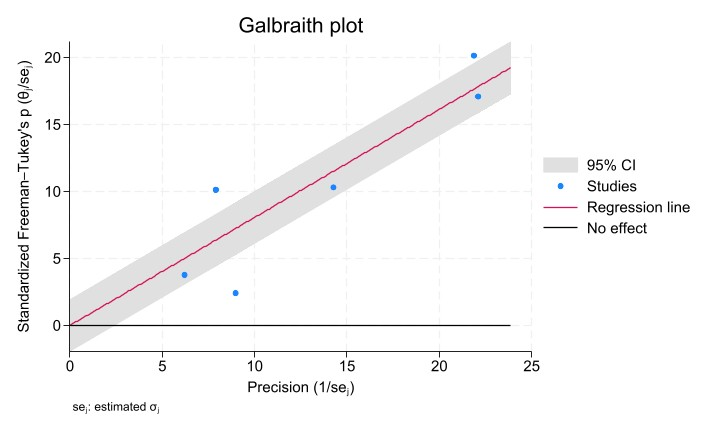

Supplement: Supplementary file 10 — Figure S9: Galbraith plot. [file JVH-33-0-s003.tiff]

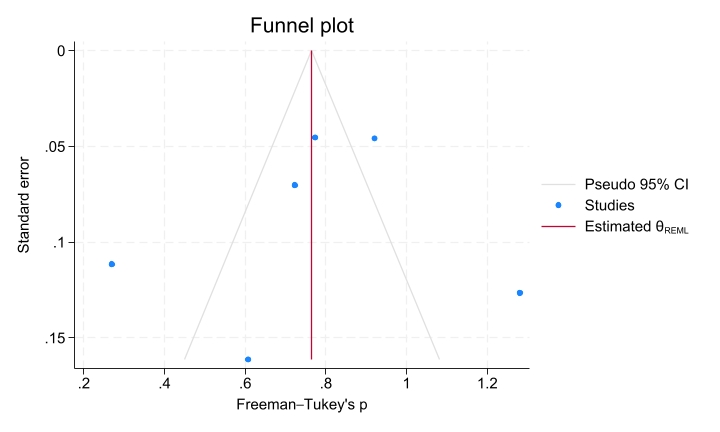

Supplement: Supplementary file 11 — Figure S10: Funnel plot; no apparent asymmetry is observed, and no imputed studies are detected applying trim‐and‐fill analysis. [file JVH-33-0-s011.tiff]
