# Supplementary material for: Hepatitis E Infection in Patients With Inflammatory Bowel Diseases: A Systematic Review and Meta‐Analysis
Source: J Viral Hepat. 2026 Feb 20;33(3):e70152. doi: 10.1111/jvh.70152 (PMC12923650; doi:10.1111/jvh.70152)
Supplement: Supplementary file 12 — Table S1: Quality assessment of the included studies. [file JVH-33-0-s004.docx]

**Supplementary Table 1.** Quality assessment of the included studies.

| Study | Q1 | Q2 | Q3 | Q4 | Q5 | Q6 | Q7 | Q8 | Overall appraisal |
| --- | --- | --- | --- | --- | --- | --- | --- | --- | --- |
| Senosiain, 2015 | N | Y | Y | Y | Y | Ν | Y | Y | Acceptable |
| Garrido, 2019 | Y | N | Y | Y | Y | Y | Y | Y | Acceptable |
| Hoffmann, 2020 | Y | Y | Y | Y | Y | Y | Y | Y | Acceptable |
| Grigas, 2021 | Y | Y | Y | Y | Y | Y | Y | Y | Acceptable |
| Kounis, 2023 | Y | Y | Y | Y | Y | Y | Y | Y | Acceptable |
| Santos, 2023 | Y | Y | Y | Y | N | N | Y | Y | Acceptable |
